# Genetic Testing in Prion Disease: Psychological Consequences of the Decisions to Know or Not to Know

**DOI:** 10.3389/fgene.2019.00895

**Published:** 2019-09-20

**Authors:** Mathias Schwartz, Jean-Philippe Brandel, Marie Lise Babonneau, Christilla Boucher, Elodie Schaerer, Stephane Haik, Jean Louis Laplanche, Marcela Gargiulo, Alexandra Durr

**Affiliations:** ^1^APHP, Department of Genetics, University Hospital Pitié-Salpêtrière, Paris, France; ^2^Cellule nationale de référence des maladies de Creutzfeldt-Jakob, APHP, University Hospital Pitié-Salpêtrière, Paris, France; ^3^Institut du Cerveau et de la Moelle épinière (ICM), AP-HP, Inserm, CNRS, University Hospital Pitié-Salpêtrière, Sorbonne Université, Paris, France; ^4^Département de Biochimie et biologie moléculaire, Lariboisière Hospital, Paris, France; ^5^Laboratoire de Psychologie Clinique et Psychopathologie, Institut de Psychologie, Université Paris Descartes, Sorbonne Paris Cité, Paris, France; ^6^Institut of Myologie, University Hospital Pitié-Salpêtrière, Paris, France

**Keywords:** *PRNP*, presymptomatic testing, prion, psychological scales, anxiety

## Abstract

**Purpose:** Presymptomatic testing for susceptibility to genetic prion diseases is often delivered in difficult circumstances, as the index case is often dying when a genetic diagnosis is obtained. Since test requests in these diseases are very rare, the factors underlying decisions of relatives to be tested or not and the long-term psychological consequences are not reported.

**Methods:** We contacted subjects who had consulted between 2004 and 2017 because a relative carried a pathological *PRNP* variant. Standardized psychological scales and semistructured interviews were proposed.

**Results:** We did contact 19 of the 30 subjects who had consulted: 6 of 10 who did not undergo testing, 10 of 12 noncarriers, and 3 of 8 mutation carriers. Anxiety rates were high and similar between noncarriers and untested subjects.

**Conclusions:** Living in a family with inherited prion disease produced psychological burden, regardless of the decision to undergo testing and its results. Decisions in favor of being testing did not allow relief of anxiety about the family disease. The dilemmatic decision not to know remained a burden to be coped with. Genetic counseling procedures should take into account all these situations, even that of noncarriers and that of untested.

## Introduction

Pathogenic germline variants of the *PRNP* (prion protein) gene are responsible for 10% to 14% of prion disease (Will et al., 1998; Ladogana et al., 2005; Minikel et al., 2016). These mutations increase the risk of prion protein misfolding (Will et al., 1998; Prusiner, 2006). The core phenotypes of genetic prion diseases are genetic Creutzfeldt-Jakob disease (gCJD), Gerstmann-Sträussler-Scheinker syndrome (GSS), and fatal familial insomnia (FFI) (Klug et al., 2013; Takada et al., 2017; ). In gCJD first symptoms typically occur between ages 30 and 50 years, although a few individuals present before age 30 years or as late as the upper 80s. First signs are memory impairment and confusion, followed by ataxia and myoclonus. The course from onset to death ranges from a few months to 5 years. At the end stage of disease, the individual is generally bedbound, mute, and akinetic except for myoclonic jerks. In GSS, cerebellar dysfunction and mild dysarthria typically occur in the fourth to sixth decade. Other features are a pyramidal spasticity, an extrapyramidal bradykinesia, an increased muscle tone with or without cogwheeling, and a masked facies. Fatal familial insomnia first causes an insidious or subacute insomnia, with a worsening reduction in overall sleep time, in the fifth or sixth decade. A disturbance in autonomic function then emerges: elevated blood pressure, episodic hyperventilation, excessive lacrimation, sexual and urinary tract dysfunction, and/or a change in basal body temperature. Signs of brain stem involvement including decreased ability to gaze upward, double vision, jerky eye pursuit movements, or dysarthric speech may also appear. Over the next few months, individuals develop cerebellar ataxia. A fourth *PRNP*-related syndrome with amyloid angiopathy is typically caused by truncating mutations (Revesz et al., 2009). Otherwise, the correlation between genotype and phenotype remains a matter of debate (Takada et al., 2017; Kim et al., 2018). However, genetic databases have increased our understanding of the incomplete and age-dependent penetrance of these mutations (Check Hayden, 2016; Minikel et al., 2016). Minikel et al. (2016, 2019) showed that the most frequent pathogenic *PRNP* variants such as Pro102Leu, Asp178Asn, and Glu200Lys are fully penetrant with a large variance in the age at disease onset for the latter. Other variants, such as Val210Ile or Val180Ile, have much lower penetrance, around 10% and 1%, respectively. Even with fully penetrant mutations, index cases do not always have family history of prion disease, mostly because of the possible late onset of Glu200Lys expression (Minikel et al., 2016). Indeed, about half of all gCJD diagnoses occur in patients with no known familial history, and thus late-onset cases or unexpressed mutations are possible (Kim et al., 2018). In cases without familial knowledge about the disease, genetic diagnosis is accompanied by news of both a terrible prognosis for the patient and of a risk of relatives carrying a highly penetrant mutation, but also the possibility of nonmanifesting disease. Pretest counseling is of particular importance for these cases as a sporadic etiology could have been first suspected (Goldman et al., 2004).

The question of presymptomatic genetic testing therefore arises in difficult circumstances. The reasons behind requests from individuals for genetic testing must be thoroughly assessed, *via* a process similar to that used for Huntington disease (HD) (Bechtel and Geschwind, 2013). At-risk persons can choose whether or not to take the test within a multistep and interdisciplinary counseling framework, including geneticist, psychologist, genetic counselor, social worker, and nurses, before and after blood sampling and testing (Gargiulo et al., 2009; Gargiulo et al., 2017). Even with the possibility of prenatal or preimplantation genetic diagnosis (PGD) (Uflacker et al., 2014), genetic testing raises particular issues because it provides information not only about the person tested, but also about a risk of transmission to progeny. Only a minority of at-risk subjects decide to undergo testing (Paulsen et al., 2013; Owen et al., 2014). Contrary to HD, and probably because of its rarity, psychological impact of presymptomatic testing in genetic prion diseases has been little studied (Paulsen et al., 2013). In other young-onset dementias, testing can reduce anxiety, but may also lead to depression (Goldman, 2015). In HD, we observed after the genetic results a higher score of depression and less anxiety. The reduction of anxiety is the result of the cessation of uncertainty about the genetic status, a source of anxiety and anguish. Reasons leading a subject to not perform a presymptomatic test are, however, not reported, even for less rare genetic diseases. As part of the clinical follow-up, we contacted all subjects who had come to a presymptomatic test consultation, regardless of their choice to undergo the test or not. We wanted to give them an opportunity to explain their feelings about the test and to better understand specific issues raised by *PRNP*-related disease. We investigated the personal reasons behind this decision and the long-term consequences. This analysis should help healthcare professionals to provide better guidance to individuals consulting in these difficult circumstances.

## Subjects and Methods

### Presymptomatic Testing Procedure

**Figure 1:**
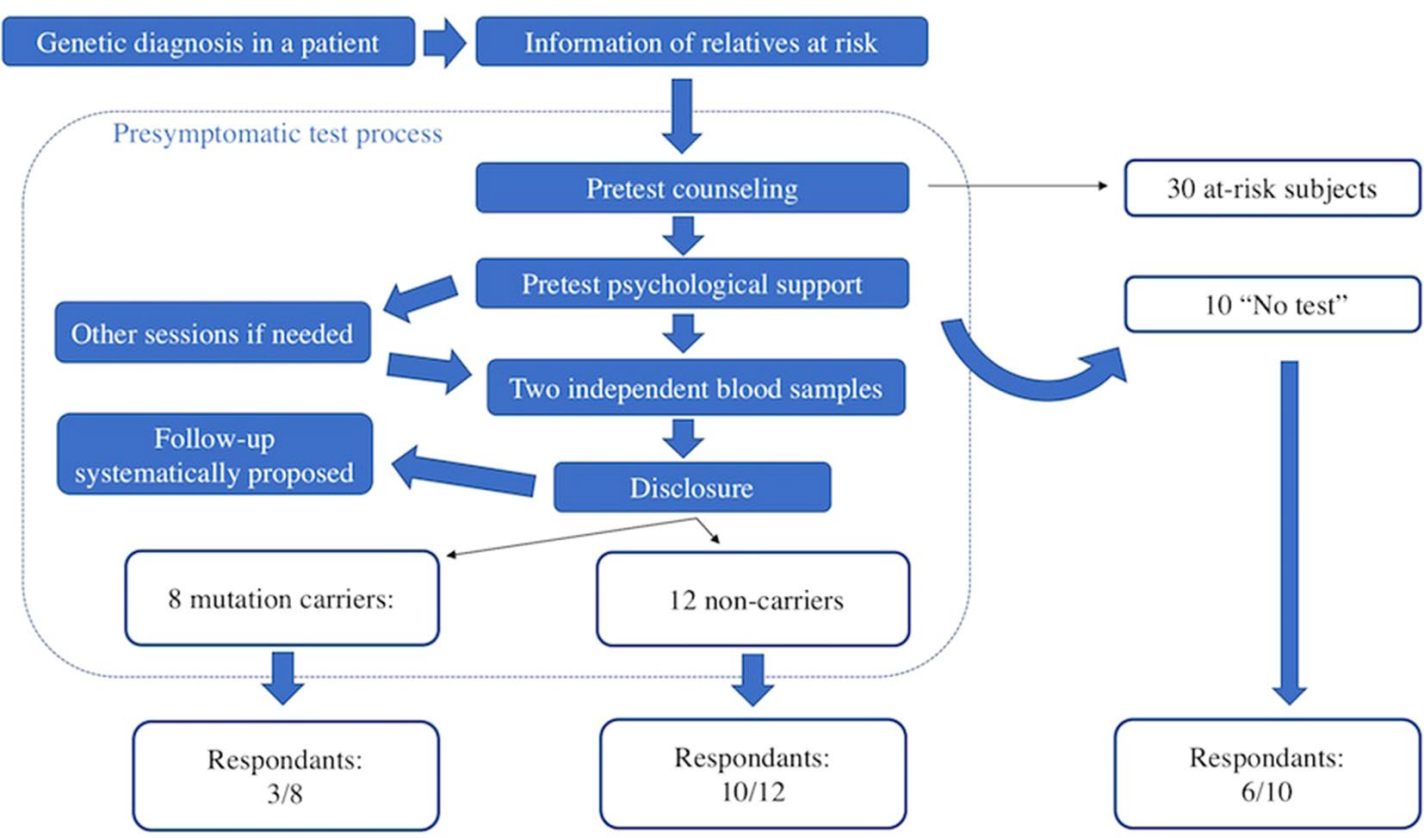
Presymptomatic test process and survey response rate.

Between 2004 and 2017, 30 subjects consulted the genetics department of Pitié-Salpétrière University Hospital in Paris after being informed that a relative had been diagnosed with a genetic prion disease. For comparison, during the same period, 950 persons at risk of HD consulted. All were asymptomatic at the time of first contact with us. The presymptomatic testing procedure is multidisciplinary (**Figure 1**). Subjects at risk and the geneticist signed an informed consent form before genetic analysis, as required under French law. The presymptomatic testing procedure and the genetic team in charge of it are submitted to the French Ministry of Health. Sanger sequencing of the *PRNP* whole coding sequences was performed from two independent blood samples, as previously described (Peoc’h et al., 2012). The results of testing were delivered at a dedicated consultation 1 month later. Psychological support was provided during testing: at least one session was required, and follow-up was systematically proposed.

### Surveys

We contacted all 30 subjects by phone in 2017 and proposed to perform semistructured interviews based on a series of multiple-choice and open questions, specific for mutation carriers, noncarriers, and those who did not undergo testing. We then tried to identify global patterns describing how the disease and the testing procedure had impacted these subjects, in order to provide better guidance to upcoming consulting at-risk individuals. Subjects could choose between a face-to-face interview and a telephone interview. Participants were informed that data would remain anonymous. According to French law, as this study relies only on surveys and interviews, it does not fall within the scope of the “Loi Jardé” (March 2012, application decree 2017-884 published in French Official Journal on May 10, 2017). It therefore does not require specific approval by an ethics committee. Written consent forms were signed by all participants. Our goal was to provide an exploratory assessment of the most salient preoccupations and the most frequent responses emerging in the context of presymptomatic testing. Several aspects were investigated: 1) global benefits or drawbacks of the presymptomatic procedure, 2) reasons leading to perform or not the test and potential doubts or regrets about this decision, 3) impact on relationship with relatives, 4) consequences of the procedure on the life course (full questionnaire, translated from French, is available in **Supplementary Data**). After the interview, the possibility of an appointment genetic counselor or psychological interview in our team was provided.

We used two standardized psychological scales: the Beck Depression Inventory (BDI) and the State-Trait Anxiety Inventory: STAI-YA for “state anxiety” (i.e., emotional reaction at the precise moment of the survey) and STAI-YB for “trait anxiety” (i.e., level of anxiety during everyday life) (Beck et al., 1961; Gaudry et al., 1975). The person is considered to be depressed if the global BDI score obtained exceeds 3, and the depression is severe if the score exceeds 15. Anxiety is considered significant if the STAI score exceeds 35, and high if greater than 55. We did not have baseline anxiety and depression levels allowing a comparison over time. We only tried to give a snapshot of feelings reported by these subjects.

## Results

### Cohort Description

Among the 30 subjects, 73% had a familial history of gCJD, 17% of FFI, and 10% of GSS. The mutations of the *PRNP* gene (NM_000311.4) identified were c.598G > A, p.Glu200Lys (nine families with gCJD); c.532G > A, p.Asp178Asn (two families with FFI and the Asp178Asn-129Met haplotype and one gCJD family with the Asp178Asn-129Val haplotype); c.631G > C, p.Glu211Gln (one gCJD family); c.305C > T, p.Pro102Leu (one GSS family); and c.250_251ins192 corresponding to eight octapeptides repeat insertion (two GSS families). One *PRNP* testing was made without any prior knowledge of the underlying mutation based on the familial transmission of the disease and the homogeneity of the clinical picture.

**Figure 2:**
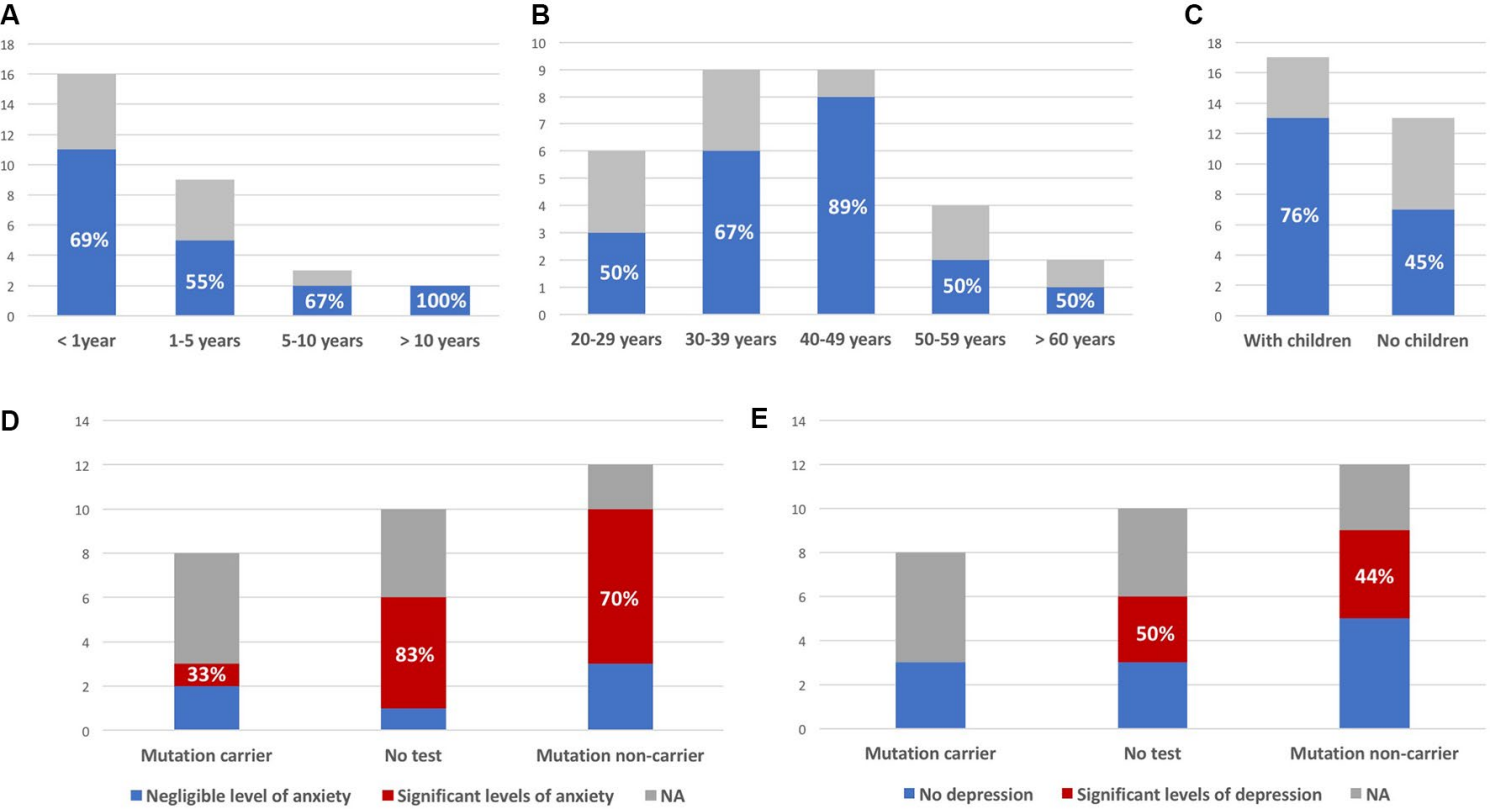
Interval between being told of risk and first consultation **(A)**, age at first consultation **(B)**, and parental status at first consultation **(C)**; blue (colored version) or black (black and white version) bars represent the proportion of at-risk individuals deciding to undergo presymptomatic testing (indicated as percentages, all observed differences between the groups were not statistically significant). **(D)** Number of subjects with significant levels of anxiety (State-Trait Inventory score above 35); similar results were obtained for “state anxiety” and “trait anxiety.” **(E)** Number of subjects with significant levels of depression (Beck Depression Inventory score >3). *Y* axis: number of patients; NA: subjects not responding to the survey.

Mean age at first contact was 40 years (range, 21–65 years). The interval between the subject being informed of the risk and attending a first consultation ranged from a few weeks to 20 years. Most subjects consulted within a few months of learning of the risk (**Figures 2A, B**). Overall, 20 (67%) of the 30 at-risk individuals decided to undergo testing (including 13 of the 17 subjects with children, **Figure 2C**), and eight were found to carry the familial mutation. The principal reasons for requesting testing were to inform children of a potential risk, to be aware of the risk of transmission before deciding to have children, or more globally to organize their lives and anticipate the consequences of disease. Other subjects invoked the impossibility of living in a state of doubt and a legitimate “right” to know.

### Response Rate

Among eight mutation carriers, three were impossible to reach (last contact between 7 and 9 years ago), and two refused to answer. Only three agreed to answer, one with some reluctance and another reported felling “stressed” by our phone call. The response rate was higher for noncarriers (10/12: two impossible to reach) and for subjects who did not undergo testing (6/10: three impossible to reach and one asking for being contacted later because it was not the right time to “think about that”). Noncarriers and untested subjects often expressed gratitude for being contacted, and none expressly refused to answer, in contrary of two of the eight mutation carriers. One untested seized this opportunity to perform the test and did not carry the mutation. Of the 19 individuals agreeing to participate in this study (three mutation carriers, 10 noncarriers, and six untested), four were interviewed face-to-face (one mutation carrier and three untested); 14 chose a phone interview, and the last one fulfilled questionnaires on his own and sent them to us by postal mail.

### Scores of Depression and Anxiety Assessment

Measured levels of anxiety were elevated, with no relevant difference between results for the STAI-YA and STAI-YB scales (38.5 ± 10.7 and 39.8 ± 10.6, respectively). They were not correlated to time since last consultation (anxious: 6.3 ± 3.2 years; nonanxious: 4.7 ± 3.4 years; *p*-value = 0.35). Among six untested individuals, five had a score greater than 35, and among 10 noncarriers, seven had significant levels of anxiety (83% and 70% respectively, **Figure 2D**). The variability of reported experiences in the three mutation carriers and their low number do not allow to compare them to the other two groups. Among them, only one had significant anxiety (STAI-YA = 42; STAI-YB = 39). Similar results were obtained on the BDI scale (**Figure 2E**). Half (3/6) of the untested subjects and 44% (4/9) of noncarriers had significant levels of depression. One of the noncarriers did not complete the BDI survey because she found it too intrusive (her STAI-YA score was 62). None of the three mutation carriers were depressed.

### Qualitative Assessment by Semistructured Interviews: Initial Information and Consultation Experiences

Semistructured interviews provided much more information about psychological outcomes, including feelings of ambiguity about the utility of the testing procedure. Subjects reported that “finding contacts,” in a place where genetic testing is “not taken lightly” was “reassuring” and “helped to move forward.” Despite efforts from the team to provide adequate psychological support, some at-risk persons retrospectively criticized the usefulness because “too little is known” about the disease and its treatment or because “a 50% risk doesn’t mean much” to them. However, knowledge of the risk had a dramatic impact on one individual, who decided to “get married faster.” Another person said that the procedure “forced him to look for information” and to “reappropriate” his father’s death. Others described the whole procedure as a “loss of time,” mostly because they had “already decided” to undergo testing. Paradoxically, one individual reproached that testing was performed in “harsh circumstances” but planned to be tested abroad, to avoid multistep counseling procedure as proposed by our team and thus “go faster.”

In families with longer histories of disease, more precise information that it would be “difficult to communicate” to the family (50% risk, possibility of testing) was considered useful. The procedure helped some people to “prepare” themselves and their “loved ones” to “get ready.” In cases of more recent diagnosis of the index case, often while he was dying, this information was seen in a harsher light. Subjects said that even though it was “a good thing to know the truth,” this “violent information” signified a “loss of hope” and was like a “live grenade given in their hand.” One person reported an “earthquake in the family” causing “discord” and “stupefaction” and “murderous statements,” with one cousin believing “she will die within 5 years.” Another difficult situation was that of an individual who learned of her risk through a phone call from her sister-in-law, when her brother died. This led her to believe that she was also about to die, and she wanted to prepare her own death. In another family, the initial diagnosis was obtained while the index case was unconscious. The relatives said they would have preferred not knowing that they were at risk. Several patients regretted not being contacted within a few months after the first consultation.

### The Choice to Take or Not to Take the Test

Disease was frequently present in the minds of those who decided not to undergo testing. The main issue relating to testing was what to do with the information obtained. One said that she would have been tested if she did not have children. Another patient did not know how to inform her children of a bad result without it “rotting their life the way it rotted” hers. The only person at-risk certain that she would never be tested was already reassured because of her age and because she did not have children. Another person assumed that he carried the mutation and that testing therefore “wouldn’t change his life.” Testing would “give meaning” to current PGD procedures, but he would undergo testing only if PGD failed. Two others did not know whether they might “one day be ready to hear a bad result,” which would worsen mourning or “kill any motivation.” They said they would undergo testing “only if there is a hope,” such as a trial of preventive treatment.

The three mutation carriers had very different feelings about testing. One said it was “easier to know before the symptoms occur.” The second had “mixed” feelings about the benefit, but would take the same decision today because it was “impossible to live in doubt.” The third said that he “should have remained in doubt” and that testing was globally “deleterious.” He still had doubts about the penetrance in his family.

Among the 10 noncarriers, the principal reasons for undergoing testing cited were to inform children or to “say goodbye” to them or to satisfy a “huge need to know” about the risk of transmission. Several people said that they would not have children with a 50% risk of transmitting the mutation. The decision, however, created “huge anguish.” Upon learning that they were noncarriers, almost all subjects reported a feeling of “liberation” from a “huge burden.” Testing was seen as providing a “great chance” allowing to be better “respected as thinking beings.” It gave “a reason to move forward” and helped to “accept the disease.” However, the feeling of relief had major limits. A “remaining burden” for relatives was a common concern. It was “impossible to forget the disease and the death throes” of relatives. Feelings of injustice, anger, and revolt were reported. Testing created “new questions” like “why me?” Two people found it difficult to believe their results. One thought she had been given a good result purely to reassure her. The other was still worried that a mistake might have occurred, so there was “always a doubt.”

### Fear of the Disease, Feel of Guilt, and Self-Observation

In mutation carriers, fear of the disease seemed to be even stronger than fear of death, and one subject raised the possibility of “committing suicide to avoid the death throe.” Daily fears were also reported in at-risk individuals who had not undergone testing. A noncarrier reported a depressing feeling of being the “last survivor.” Indeed, even in noncarriers, this “worse than torture” disease was never fully forgotten.

A very common feeling was the urge to “live life to the fullest.” This need to “enjoy life” was spontaneously reported by all three mutation carriers, 1 of 6 subjects who did not undergo testing, and 4 of 10 noncarriers. In noncarriers, the feeling of having “avoided tragedy” triggered “dramatic changes in life perception.”

One of the most frequently reported features, regardless of mutation status, was self-observation behavior. A fear of an increase in self-observation may be one of the reasons underlying the decision not to undergo testing. Indeed, one of the mutation carriers had been certain that she was sick for several years. Another person was unable to sleep while awaiting the result and feared it was a first sign of IFF. Another saw the disease in pain related to a sciatic nerve, which was subsequently put down to her anxiety. Being a noncarrier did not necessarily stop self-observation behavior: one, who did not question the genetic results, could not refrain from looking up the symptoms, and several reported not being able to stop themselves looking for symptoms in at-risk relatives.

### Outcomes on Procreation Projects

One mutation carrier was still considering having children naturally, but PGD and prenatal testing were seen as the “only remaining hope” for those who had not undergone testing. This “reappropriation of control” was often cited as a reason for undergoing testing in the future. These procedures could also cause arguments in families with some relatives seeing it as “eugenics.”

Spouses played a major role in the testing process and not only because of parenthood plans. The possibility of a bad result was sometimes less well anticipated, or more feared, by them than by person at risk. The spouse was reluctant about testing in 2 of 6 individuals who did not undergo testing. In one of these cases, even talking about testing was impossible. Some of the at-risk individuals underwent testing despite the reluctance of their spouses. However, the doubt was harder to accept for the spouses of two at-risk individuals who were finally found not to carry the mutation. In these two cases, testing “revived” their relationships, making them more “mature,” making daily troubles seem “less important,” and allowing the couples to make new plans.

### A Taboo About Familial Disease

In several families, the disease was described as a “taboo.” Relatives did not always know that they were at risk, the people who underwent testing did not always say that they had done so, and some even concealed the results. Dealing with the information seemed to become increasingly complicated with increasing family size. In several families, divisions were noted between people, according to their knowledge and opinion of their risk. Some did not want to inform their relatives, to prevent “useless worrying.” Some people were, on the other hand, afraid of their relatives’ reaction to learning that the risk had been hidden from them. Subjects were waiting not only for the development of symptoms in themselves or their relatives, but also for the “critical moments” in which family secrets are revealed, with “an avalanche of consequences for the next generation.” Even in situations in which the risk was known, discussions about the relevance of testing could become a source of arguments. One subject reported that “talking a lot” had been “enriching” and had “tightened links.” However, this case was an exception, as these subjects were usually avoided.

Sharing results was often a complicated issue. A “noncarrying” result was usually shared with widowed parents, to reassure them. A “mutation-carrying” result was not communicated to the parents to avoid triggering worry or “depression.” The total opposite pattern of behavior tended to be adopted with siblings or more distant relatives, with whom it seemed easier to talk about a “mutation-carrying” result, to let them know that they were “not alone.” A “noncarrying” result was more difficult to share. The feeling that such results were “taboo” led to some people concealing their favorable results, sometimes causing siblings to lose touch with each other.

### Informing Children

Even though wanting to inform children is one of the potential reasons for being tested, neither of the two mutation carriers who had children had informed them. It was a source of internal struggle, as one could not accept the idea of the children learning about their risk from someone else. For two other subjects with children about 20 years old, not knowing how to tell them about a potential bad result was the principal reasons for not undergoing testing yet. Noncarriers found it easier to inform their children, even if the children were very young. In 5 of 7 cases, the children were informed after testing. The youngest child informed was 4 years old, and several others were younger than 10 years.

### Fear of Contagion and Stigmatization

There is no evidence that gCJD is spread through ordinary day-to-day contact with those affected or by airborne droplets, blood, or sexual contact. However, and despite the fact that this information has been given during the test procedure, most subjects felt that their family history “should not be revealed to everyone” as “people might not understand.” Families felt “abandoned,” and the lack of national patients’ associations was deplored by some. A “fear of stigmatization” was also reported. One subject saw this “taboo disease” as a “secret” she had to “hide.” Several people did not inform their doctors about their risk because “it would not change anything” or because they feared a loss of access to some types of care. Indeed, one patient was refused varicose vein surgery because of the risk, until the case was reported to French antidiscrimination authorities. One patient with a favorable testing result regretted she was still refused for blood donation, as well as her children. She hoped her good result would change that, but blood donation is forbidden for all family members of prion disease cases, regardless of an identified genetic cause. Various fears about contagion were reported: during dental care or meningioma surgery, or even during sexual intercourse or after a cut sustained while cooking.

## Discussion

In this study, we investigated the impact of being at risk of genetic prion diseases, and the reasons for and long-term consequences of genetic testing. About 65% of the individuals at risk, and who consulted at least once, decided to undergo testing, a proportion very similar to that reported for HD (Gargiulo et al., 2017). Main limitations of this study were that genetic prion diseases are very rare, and therefore the number of subjects included was low. Consequently to the rarity of the disease, we wanted to explore personal feelings about the disease and the testing procedure, with qualitative data analysis. We used the STAI and BDI scales to obtain some quantitative assessment of the participants’ states of mind, but these two scales cannot describe the highly complex feelings reported. We could not compare the levels of individual anxiety and depression before and after the testing, but only at the time of our questionnaire. Anxiety was high in all three groups: at-risk individuals who did not undergo testing, mutation carriers, and noncarriers. High level of anxiety in subject who did not undergo testing is not surprising as predictive testing is usually done in a self-selected group of people who think they can cope, as proposed in HD (Decruyenaere et al., 1995). The lack of difference between state and trait anxiety scores suggests that interviews were not seen as a specific source of stress. The fact that most interviews were carried out by phone could have influenced the answers to the questions, but since each life story is very unique to the person at risk, we are not able to evaluate the impact of the format.

We compensated the limitations by giving the time to at-risk subjects to describe in a very detailed way their feelings about this genetic disease. We could define global patterns that we think can help to understand their state of mind and can have practical consequences.

Our first main result was the difficulty to contact mutation carriers. Although this study occurred 7 to 9 years after testing, these subjects were unlikely to have developed symptoms, because they were not reported in the national file of patients with prion diseases. Those we were able to contact were frequently reluctant. We did not anticipate this phenomenon, and indeed, we expected these subjects to request follow-up. Most of the individuals complaining of the lack of follow-up were not mutation carriers. Instead, mutation carriers seemed to be trying to live their lives actively without thinking about it.

Issues reported by noncarriers emphasize that the genetic diagnosis is made on a whole family and not only at-risk individuals. As the whole family is “affected,” pretest counseling could be improved in two aspects. First, we should insist more before the test that a good result will not release from some difficulties the subject will have to cope with. Even if a “noncarrying” result is always a huge relief, especially for children-related fears, the disease is never forgotten, and the test should not be performed in this perspective. Second, and even more importantly, more precautions should be taken at the time of the initial diagnosis in the family. The more complicated familial situations reported occurred when the index case was diagnosed while dying and without the whole family realizing the impact of such a diagnosis. Indeed, a case has been reported where after a discussion with the family member, these latters decided they prefer to not know if the index case was genetic or sporadic (Goldman et al., 2004).

In conclusion, decisions in favor of being testing did not allow relief of anxiety about the family disease. The dilemmatic decision not to know remained a burden to be coped with. Genetic counselors should take into account all situations, even that of noncarriers and that of untested.

## Data Availability

The raw data supporting the conclusions of this manuscript will be made available by the authors, without undue reservation, to any qualified researcher.

## Ethics Statement

According to French law, as this study only relies on surveys and interviews, it does not fall within the scope of the “Loi Jardé” (March 2012, application decree 2017-884 published in French Official Journal on May 10th 2017). It therefore does not require specific approval by an ethics committee. Written consent forms were signed by all participants.

## Author Contributions

MS performed most of the interviews and wrote the manuscript. J-PB referred most index patients and edited the manuscript. MB and CB performed psychological sessions. ES was involved as genetic counselor. SH and JL performed molecular sequencing of the *PRNP* gene and edited the manuscript. MG performed and coordinated psychological sessions and coordinated manuscript drafting. AD was in charge of all medical genetics consultations and coordinated manuscript drafting.

## Conflict of Interest Statement

The authors declare that the research was conducted in the absence of any commercial or financial relationships that could be construed as a potential conflict of interest.
