# Supplementary material for: Genetic Testing in Prion Disease: Psychological Consequences of the Decisions to Know or Not to Know
Source: Front Genet. 2019 Sep 20;10:895. doi: 10.3389/fgene.2019.00895 (PMC6764331; doi:10.3389/fgene.2019.00895)
Supplement: Supplementary file 1 [file Table_1.docx]

Annex

Madam, Sir,

This questionnaire we propose is part of the follow-up of persons at-risk during the genetic counselling procedure. The objective is to better understand their life with the genetic disease that runs in their family and for those who have undergone genetic testing (whether they are carriers or not) to learn about their experience. This study is under the responsibility of Pr. Alexandra Durr and Marcela Gargiulo of the Department of Genetics (Pitié-Salpêtrière Sorbonne University Hospital in Paris) and the ICM (Institut du Cerveau et de la Moelle épinière).

The information collected in this questionnaire is strictly confidential and will not be included in the medical file. They will be used for analytical purposes to better understand outcome and improve patient care. Persons allowed to access the data are mandated by the Professor Alexandra Durr.

**You have requested genetic counselling for a family disease,**

A) Did the various sessions were useful to understand the consequences and possible outcome on your life?

B) At the time of the first counselling session, who did you inform about your project?

C) Would you recommend this approach to another person at risk than yourself?

D) What do you think are the advantages and disadvantages of taking a pre-symptomatic genetic test?

**If you have not completed the genetic test, please answer the following question:**

E) You have chosen not to go any further in the pre-symptomatic diagnostic procedure, do you think this decision may change in the future and why?

**The following questions are only relevant if you did take the test**.

1) The decision to request this test was made by:

2) Who were the people informed of your decision to ask for testing?

3) With regard to the length of the test procedure, did you have the impression that it was: Very long/Long/Long enough/Not long enough?

4) What was your state of mind waiting for the result?

□ You were anxious

□ You were very worried

□ You were a little worried

□ You were confident

5) Have you ever had any doubts about your decision to take the test? (if yes, could you explain why?)

6) Following the result, do you think your life has changed?

7) Are you satisfied that you asked for it?

8) Who were the persons notified of your test result? (Please specify at which time)

9) After the result, did you think at the time that the relationship with your family changed?

10) Today, do you think that your relationship with your family did change after the result of your test?

11) Do you think that this test has helped you (several possible answers)

□ To think about the disease

□ To understand it

□ To accept it

□ To turn the page

□ To hate the disease

□ To forget about the disease

□ Other

12) How do you feel since the test result? (several possible answers)

□ Relieved

□ Anxious

□ Sad

□ In revolt

□ Indifferent

□ Concerned

□ Peace of mind

□ Pleased

□ Satisfied

13) In relation to your health, how do you feel?

□ In good health

□ Unhealthy. Specify:

□ Other:

14) As far as the counselling team is concerned, you had the impression:

□ Not being able to count on the them

□ To be divided about them

□ To be able to rely on them

□ Not to need to rely on them

15) Do you see anything that seems important to you that was not asked in this questionnaire?

**The following questions are only relevant to those who have been tested and who are carriers of a mutation**

1) How did you first feel knowing you were carrying the gene mutation?

2) What means or strategies have been most beneficial to you during this period?

3) How do you currently experience knowing that you are a carrier of the gene mutation?

4) How does the result influence your daily way of life?

5) Do you believe that your situation requires medical and/or psychological follow-up?

6) Do you have regular medical follow-up?

7) Since then, have you consulted a doctor on an occasional basis?

8) Do you feel that you are more attentive to the first signs that could be the beginning of the disease?

9) Do you think you have any symptoms of the disease?

10) Do you have the impression that your family and friends are more attentive to the first signs that could be regarded as part of the beginning of the disease?

11) Have there been any major life events since then?

□ Birth event

□ Wedding

□ Separation/Divorce

□ Diploma and education award

□ Promotion or professional dismissal

□ Other, specify:

If so, do you think your status as a carrier has influenced these?

12) How do you perceive the fact of having taken this genetic test with regard to the impact on your life?

13) Do you have any regrets about your decision to take the genetic test?

□ Yes

□ No

□ I don’t know

14) If you could go back in time, would you make the same decision about the testing?
